# Prenatal stress, intimate partner violence and maternal cortisol trajectories: insights from a prospective birth cohort from São Paulo, Brazil

**DOI:** 10.1038/s41598-026-50811-9

**Published:** 2026-05-04

**Authors:** Lukas Blumrich, Mark V. Flinn, Luis Augusto Rohde, Guilherme Vanoni Polanczyk, Eurípedes Constantino Miguel, Sandra Josefina Ferraz Ellero Grisi, Helena Brentani, Alexandre Archanjo Ferraro

**Affiliations:** 1 Department of Pediatrics, Faculty of Medicine, University of São Paulo, São Paulo, Brazil; 2 Department of Anthropology, Baylor University, Waco, USA; 3 ADHD Outpatient Program & Developmental Psychiatry Program, Hospital de Clinicas de Porto Alegre, Federal University of Rio Grande do Sul, Medical Council UniEduK, National Institute of Developmental Psychiatry & National Center for Innovation and Research in Mental Health, São Paulo, Brazil; 4 Department of Psychiatry, Faculty of Medicine, University of São Paulo, São Paulo, Brazil

**Keywords:** Cortisol, Hair cortisol, Pregnancy, Violence, Prenatal stress, Cohort, Biomarkers, Diseases, Endocrinology, Medical research, Physiology, Psychology, Psychology

## Abstract

Prenatal stress has been implicated in alterations of maternal hypothalamic–pituitary–adrenal (HPA) axis functioning, yet evidence linking psychosocial stressors to long-term cortisol biology remains inconsistent. Hair cortisol concentration (HCC) provides an integrated measure of cumulative glucocorticoid exposure, allowing investigation of whether diverse prenatal stressors shape maternal cortisol trajectories during pregnancy and postpartum. We analyzed data from a prospective population-based birth cohort from São Paulo, Brazil. Pregnant women (*n* = 185) were assessed in the third trimester for exposure to intimate partner violence (IPV), emotional responses to pregnancy, and anxiety or depression using standardized instruments (WHO-VAW, MINI). Maternal HCC was quantified from 7 cm hair segments representing the last four months of pregnancy and the first two postpartum months. We compared cortisol levels across exposure groups using t-tests and Mann–Whitney tests, and estimated longitudinal associations with linear mixed-effects models. Cortisol levels declined significantly from pregnancy to postpartum (*p* < 0.001). Most prenatal stressors—including psychological IPV, anxiety, depression, and negative emotional responses to pregnancy—were not associated with HCC at individual time points nor in longitudinal models. Acute prenatal physical/sexual IPV was associated with lower HCC at specific prepartum time points and with lower mean HCC in longitudinal analyses. In this cohort, maternal cortisol biology showed limited sensitivity to most psychosocial stressors during pregnancy. Only acute physical or sexual IPV was associated with reduced cumulative cortisol levels. These findings highlight the specificity of severe interpersonal violence as a biological stressor and underscore the complexity of interpreting cortisol as a biomarker of prenatal stress.

**Supplementary Information:**

The online version contains supplementary material available at 10.1038/s41598-026-50811-9.

## Introduction

The study of the detrimental effects of prenatal stress on the health and disease of the next generation is a growing field, as is the recognition of the breadth of its effects and its role in reinforcing inequalities^1^. Although the definition of what constitutes prenatal stress remains somewhat frail^2,3^, there is vast literature on the effects of prenatal anxiety, depression, perceived stress, hunger and exposure to traumatic experiences on offspring’s health and development^4–6^.

Glucocorticoids are implicated in the mechanisms linking prenatal stress and adverse outcomes^7,8^. Cortisol is often investigated as a biomarker^5,9^. Unfortunately, there is often a conflation between biomarker measures and mechanisms^10,11^. Cortisol is commonly treated in biomedical literature as ‘the stress hormone’, and cortisol measures are referred to as stress^12^. This conflation both excludes other mechanisms (e.g. epigenetics, changes in microbiome, inflammation) and ignores the fact that glucocorticoids are related to an enormous diversity of regulatory and metabolic processes in mammals, thus restricting scientific development on the study of stressors^13,14^.

Given the substantial intra- and inter-individual variability in cortisol levels, studies assessing its relationship with prenatal stress require careful contextualization^15^. Numerous studies have explored cortisol dynamics during pregnancy, but the evidence linking prenatal stress to altered cortisol biology remains inconclusive^16,17^. While some studies suggest an association between elevated cortisol and prenatal anxiety or depression, others report weak or inconsistent findings^17^. Such variability underscores the importance of methodological considerations, including the type of cortisol measure used, the timing of assessment, and individual differences in HPA axis regulation^9,16,18^.

Hair cortisol analysis offers an opportunity to assess long-term cortisol exposure^19^. Unlike saliva, serum or sweat cortisol, which capture acute fluctuations, hair cortisol is continuously embedded in the follicle matrix, providing an integrated measure of systemic cortisol levels over months. This method is relevant for studying prenatal stress, as it allows retrospective assessment of cumulative averages of cortisol during pregnancy^17,19^.

Recent reviews have highlighted substantial heterogeneity in the associations between prenatal stress and maternal cortisol levels, with findings varying depending on the type, severity, and timing of stress exposure as well as the methods used to assess cortisol^17,20–22^. In particular, relatively few studies have examined multiple forms of prenatal stress simultaneously, making it difficult to determine whether distinct stressors—such as intimate partner violence, emotional responses to pregnancy, or clinically diagnosed anxiety and depression—have differential associations with maternal cortisol regulation. Moreover, evidence from middle-income countries remains limited.

Prenatal stress is a multidimensional construct that can arise from different sources, including severe interpersonal stressors, subjective emotional responses to pregnancy, and clinically diagnosed mental health conditions. Intimate partner violence represents a particularly severe form of interpersonal stress involving threat, coercion, and potential physical harm^23^. In contrast, emotional reactions to pregnancy capture women’s subjective appraisal of the pregnancy experience, while anxiety and depression represent clinically defined psychiatric conditions associated with psychological distress. Although these domains may overlap, they reflect distinct pathways through which psychosocial stress may influence biological stress regulation during pregnancy.

Therefore, the present study examined the association between several prenatal stress exposures—including intimate partner violence, emotional responses to pregnancy, and the presence of anxiety or depression—and maternal hair cortisol concentrations during pregnancy and the early postpartum period in a population-based birth cohort in Brazil. By using hair cortisol as a measure of cumulative glucocorticoid exposure, we sought to assess whether different forms of prenatal stress are associated with distinct maternal cortisol trajectories.

## Methods

### Study design

This study analyses data from a prospective, population-based birth cohort^24^ from the Butantã region of the city of São Paulo. Participants were enrolled at the start of the third trimester of pregnancy and followed through to the age of 10.

### Population and sample

This study targeted pregnant women receiving care at five Basic Health Units (Unidades Básicas de Saúde, UBS) within the Butantã Health District, situated in the western region of São Paulo, Brazil. Eligibility extended to those attending prenatal appointments between July 2010 and December 2012. Each week, the first five pregnant women attending their appointments at each unit were enrolled in the study. Women with multiple pregnancies or children born with conditions that could impair growth or development were excluded from the analysis.

The Butantã Health District operates under the Family Health Strategy (Estratégia Saúde da Família) Program, which promotes prenatal care access through initiatives such as regular home visits and early pregnancy detection. This densely populated region, located on the outskirts of São Paulo, faces notable social and economic challenges, including inadequate public infrastructure and pronounced social inequalities. The population includes both lower-income households and an emerging middle class, coexisting in a setting characterized by schools, nurseries, and churches amid ongoing tensions linked to drug trafficking and law enforcement activities^24^.

All methods were performed in accordance with the relevant guidelines and regulations for research involving human participants, including the Declaration of Helsinki. The study protocol was approved by the Research Ethics Committees of the Hospital das Clínicas of the Faculty of Medicine of the University of São Paulo (CAPPesq protocol number 0054/09) and the Health Department of the Municipality of São Paulo (protocol number 122/10). Written informed consent was obtained from all participants.

### Variables

#### Prenatal stress exposures

Stress during pregnancy was conceived by means of four different exposures: occurrence of intimate partner violence (considered acute if happened only in the previous 12 months or previous in the case of previous reports), feelings about pregnancy upon discovery, and diagnosis of anxiety or depression during pregnancy. Data collection was conducted at the first interview, with a mean gestational age of 30 weeks (SD = 3.4 weeks).

#### Intimate partner violence

Information on the occurrence of violence during the preconceptional and prenatal period were obtained using the WHO Violence Against Women Questionnaire^23,25^. The WHO Violence Against Women (WHO-VAW) questionnaire has been previously validated for use in Brazil and has demonstrated good reliability and construct validity in Brazilian populations^25^. Violence during pregnancy was modeled as a binary exposure, based on the responses to the WHO-VAW Questionnaire. Due to the small number of positive cases, physical and sexual violence were pooled together in the analyses.

#### Anxiety and depression

Depression and anxiety during pregnancy were evaluated with the MINI (MINI International Neuropsychiatric Interview^26,27^ a structured diagnostic interview designed to identify DSM-IV and ICD-10 psychiatric disorders.

#### Emotional responses to pregnancy

Information on feelings about pregnancy were obtained through the question “How did you feel when you first heard you were pregnant?” and categorized as (a) predominance of positive feelings, (b) minor stress related to the discovery, (c) moderate stress related to the discovery, (d) severe stress related to the discovery and accepting pregnancy and (e) an abortion was seriously considered. For analysis, this variable was dichotomized based on conceptual grouping: categories (a) and (b) were classified as predominantly positive feelings, while categories (c)–(e) were classified as predominantly negative feelings.

#### Hair cortisol measurement

Hair samples were collected at the end of the second month postpartum, allowing for the evaluation of cortisol levels from the preceding six months, including the last four months of pregnancy and the first two months postpartum. Assuming an average hair growth rate of 1 centimeter per month, 7-centimeter hair samples collected from the base of the skull provided sufficient material for this analysis.

The hair samples were stored in labeled plastic tubes at room temperature, adhering to the laboratory’s standard protocol. They were then sent for analysis to the Specialized Laboratory for Scientific Analysis (LEAC). Each 50 mg sample underwent a two-step washing process: first, two rinses with 40 ml of water, followed by two rinses with 40 ml of isopropanol, all performed on a rotating plate at 130 rpm for 3 min. After washing, the hair was cut into small pieces using surgical scissors and placed in glass scintillation vials.

To extract cortisol, HPLC-grade methanol was added to the hair at a concentration of 100 µl/mg. The samples were subjected to ultrasonic vibration for 30 min and then incubated at 50 °C for 24 h. Following incubation, the samples were centrifuged at 3000 rpm for 30 min, and the methanol was evaporated under a nitrogen stream. For samples with less than 30 mg of hair, extract volumes were adjusted proportionally to the available hair weight (5, 10, or 15 mg). The dried extracts were reconstituted in 150–250 µl of phosphate-buffered saline (PBS) at pH 7.0 and centrifuged for an additional 90 s to ensure thorough mixing. Quantitative determination of hair cortisol levels in the extracts was performed using an enzyme-linked immunosorbent assay (ELISA) specific for salivary cortisol (Cat. # KAPDB290, Lot 150810, DiaSource). The assay was conducted following the manufacturer’s instructions. The intra- and inter-assay coefficients of variation were both below 10.3%.

### Statistical analysis

All statistical analyses were conducted using Stata (version 17; StataCorp, College Station, TX, USA). To ensure the validity of parametric tests, we first assessed the normality of continuous variables using the Shapiro-Wilk test, separately for each exposure group. A p-value < 0.05 was considered evidence of non-normality.

Next, Levene’s test for equality of variances was performed to determine whether the variance of hair cortisol concentrations differed between exposed and non-exposed groups. When variances were equal (*p* > 0.05), we applied a two-sample t-test to compare means between groups. If unequal variances were detected (*p* < 0.05), Welch’s t-test was used to account for heterogeneity in variance.

For comparisons where the normality assumption was violated, we applied the Mann-Whitney U test, a non-parametric alternative that compares the distributions of the outcome variable between groups.

To investigate the longitudinal association between exposure variables and repeated cortisol measures, we employed linear mixed-effects models (LMMs) with an interaction term between exposure and time, and using restricted maximum likelihood estimation (REML). These models accounted for within-subject correlation and individual variability by including a random intercept for each participant. Cortisol measurements were analyzed in long format, where each row represented a unique time point for each participant.

For each exposure variable, a separate LMM was estimated with cortisol as the dependent variable and exposure and time as fixed effects. Time was modeled as a categorical variable to account for non-linear changes across the six repeated measurements. A random intercept was included to allow for individual differences in baseline cortisol levels. Model selection was guided by Akaike Information Criterion (AIC) and Bayesian Information Criterion (BIC), and likelihood ratio tests (LRTs) were used to compare models with and without random slopes for time.

The proportion of missing data for study variables is presented in Supplementary Table S1. Descriptive and bivariate analyses were conducted using complete-case data for the variables included in each analysis. Longitudinal associations between prenatal violence and cortisol trajectories were examined using linear mixed-effects models estimated with restricted maximum likelihood (REML), which allows inclusion of participants with incomplete repeated measures by using all available observations under the assumption that data are missing at random.

Statistical significance was set at *p* < 0.05, and 95% confidence intervals (CIs) were reported for all parameter estimates. Postestimation margins and interaction plots were generated to visualize cortisol trajectories over time and differences between exposure groups.

## Results

Table 1 presents the demographic and psychosocial characteristics of the 183 participating mothers. Among them, 18.6% (*n* = 34) reported psychological violence, while 9.3% (*n* = 17) experienced physical violence, and 0.5% (*n* = 1) reported sexual violence during pregnancy. A diagnosis of anxiety was recorded in 12.57% (*n* = 23) of participants, and depression in 9.3% (*n* = 17). Regarding initial emotional responses to pregnancy, 43.2% (*n* = 79) reported predominantly positive feelings, whereas 4.4% (*n* = 8) seriously considered an abortion. The majority of participants were aged 20–35 years (64.8%), identified as Black (69.1%), and in lower- to middle-income categories (76.2%).

**Table 1:** Sociodemographic and psychosocial characteristics of the study sample.

Variables	Categories	*N* (%)
Violence during pregnancy	Psychological	34 (18.58)
	Physical	17 (9.28)
	Sexual	1 (0.50)
Past violence exposure	Psychological	39 (21.3)
	Physical	31 (16.93)
	Sexual	13 (7.1)
Feelings about pregnancy	Predominance of positive feelings	79 (43.16)
	Minor Stress	48 (26.22)
	Moderate Stress	21 (11.47)
	Severe Stress	24 (13.11)
	Considered Having an Abortion	8 (4.37)
Anxiety	Yes	23 (12.57)
Depression	Yes	17 (9.28)
Maternal age	< 20	34 (18.57)
	20 a 35	116 (63.38)
	> 35	29 (15.84)
Maternal skin color	Black	125 (68.30)
	Non-Black	56 (30.06)
Socioeconomic Class (ABEP Classification System)	A and B	43 (23.49)
	C	108 (59.01)
	D and E	30 (16.39)
Maternal education	0–7 years	30 (16.39)
	7–10 years	62 (33.87)
	> 10 years	87 (47.54)

Table 2 presents the unadjusted associations between prenatal stress and maternal hair cortisol at different time points. Overall, most prenatal stress exposures were not significantly associated with cortisol levels at any individual time point. However, acute exposure to physical or sexual intimate partner violence was associated with lower cortisol levels at 3 months (*p* = 0.036) and 2 months (*p* = 0.048) before birth. No statistically significant associations were observed for psychological violence, anxiety, depression, or negative feelings about pregnancy.

**Fig. 1:**
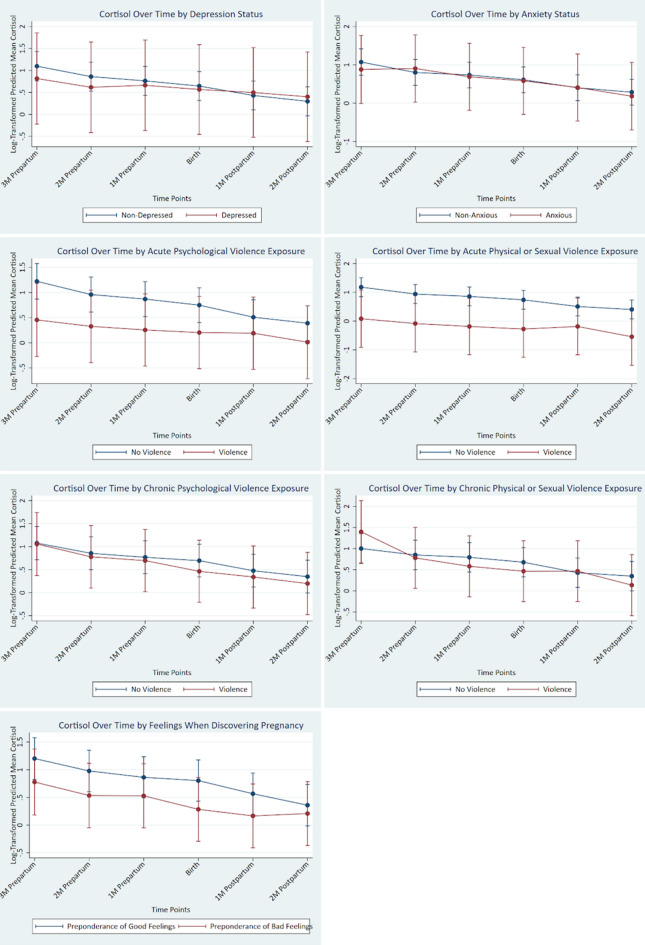
Maternal hair cortisol trajectories during late pregnancy and the postpartum period according to prenatal stress exposure. Predicted mean cortisol levels derived from linear mixed-effects models across six monthly segments, covering the last four months of pregnancy and the first two months postpartum. Lines represent estimated trajectories for exposed and non-exposed groups, illustrating the overall physiological decline in cortisol and differences associated with prenatal stress exposures.

Linear mixed-effects models (Table 3; Fig. 1) assessed the association between prenatal stress and cortisol levels over time. Cortisol exhibited a significant decline from pregnancy to the postpartum period in all of the tested models (*p* < 0.001), consistent with known physiological adaptations.

Mean cortisol levels were associated with acute prenatal exposure to physical or sexual violence (β = − 1.096, 95% CI: − 2.147 to -0.047, *p* = 0.041), but the interaction between exposure and time was not significant in the model. Cortisol trajectory was only differentially affected by prenatal exposure in the case of chronic physical or sexual violence.

**Table 2:** Associations between prenatal stress exposures and maternal hair cortisol concentration at each time point.

Exposure	*P*-values for mean differences of hair cortisol measures based on prenatal stress exposure					
	3-Month Prepartum	2-Month Prepartum	1-Month Prepartum	Birth	1-Month Postpartum	2-Months Postpartum
Feelings About Pregnancy	0.765	0.493	0.337	0.267	0.538	0.936
Anxiety	0.577	0.881	0.961	0.971	0.985	0.882
Depression	0.468	0.613	0.890	0.903	0.925	0.968
(Acute) Prenatal Psychological Violence	0.056	0.104	0.131	0.200	0.540	0.881
(Acute) Prenatal Physical or Sexual Violence	0.036*	0.048*	0.061	0.064	0.276	0.175
Lifetime Psychological Violence	0.983	0.700	0.867	0.579	0.690	0.480
Lifetime Physical or Sexual Violence	0.398	0.716	0.620	0.630	0.960	0.418

*p-value < 0.05.

**Table 3:** Linear mixed-effects model estimates for the association between prenatal stress exposures and maternal hair cortisol trajectories.

Exposure	Fixed Effect (Std. Error)	95% CI	*p*-value
Feelings About Pregnancy	-0.424 (0.360)	-1.130–0.281	0.238
Anxiety	-0.194 (0.485)	-1.145–0.758	0.690
Depression	-0.281 (0.556)	-1.370–0.808	0.613
(Acute) Prenatal Psychological Violence	-1.096 (0.536)	-2.147 - -0.047	0.041
(Acute) Prenatal Physical or Sexual Violence	-0.768 (0.413)	-1.578–0.041	0.063
Lifetime Psychological Violence	-0.019 (0.395)	-0794–0.756	0.961
Lifetime Physical or Sexual Violence	-0.397 (0.418)	-0.422–1.216	0.342

## Discussion

In this prospective cohort study, we examined the association between various prenatal stressors and maternal hair cortisol trajectories from pregnancy to the postpartum period. While cortisol levels exhibited a significant decline over time, reflecting well-established physiological adaptations during and after pregnancy, we found limited evidence that prenatal stress exposures were associated with maternal hair cortisol levels. The only notable exception was acute exposure to physical or sexual intimate partner violence, which was associated with lower hair cortisol at specific prepartum time points and in longitudinal analyses. Changes in cortisol trajectories were observed only in response to previous physical or sexual violence. These findings suggest that maternal cortisol regulation during pregnancy may be influenced by acute severe stressors but remains relatively stable in response to broader psychosocial stressors.

Another possible explanation for the lack of associations observed for other stress indicators is that hair cortisol reflects cumulative glucocorticoid exposure over several months. Psychological states such as anxiety or depressive episodes may fluctuate over shorter periods and therefore may not produce sustained changes detectable in long-term cortisol measures^28,29^. In contrast, severe interpersonal stressors such as intimate partner violence may represent more persistent and threatening experiences that could have stronger physiological consequences^30,31^. Additionally, the substantial physiological increase in cortisol during pregnancy may attenuate the detectable impact of moderate psychosocial stressors on HPA-axis activity.

The prevalence of depression (9.4%) and anxiety (12.9%) observed in our sample was broadly consistent with estimates reported in studies of pregnant women in Brazil^32–34^. Previous studies have reported antenatal depression rates ranging from approximately 8% to 25% and anxiety rates between 10% and 30%, depending on the population and assessment methods used. The prevalence of physical or sexual intimate partner violence during pregnancy in our cohort (approximately 10%) was also similar to estimates reported in Brazilian studies^23,35–37^. These findings suggest that the psychosocial profile of the present sample is broadly comparable to that observed in other Brazilian populations of pregnant women, which may support the external validity of our findings.

Cortisol plays a central role in fetal development, influencing organ maturation, inflammation, and metabolic processes^5^. During pregnancy, maternal cortisol production increases progressively, peaking in the third trimester, with an important decrease in the postpartum period^38,39^. However, dysregulation of the hypothalamic-pituitary-adrenal (HPA) axis has been implicated in adverse maternal and fetal outcomes, including preterm birth, fetal growth restriction, and postpartum depression^4,9^. Given the critical role of glucocorticoids in pregnancy, understanding how prenatal stressors modulate maternal cortisol regulation is of paramount importance.

Apart from maternal production per se, there are a number of mechanisms regulating fetal cortisol biology and exposure^40,41^. The finding that the expression and activity of 11BHSD2, a placental enzyme responsible for the conversion of cortisol to the inactive cortisone, is impaired by stress exposure contributed to the possible role of cortisol in the intergenerational transmission of health and disease^41,42^. There is solid evidence of its role in the pathways relating prenatal exposures and postpartum outcomes, and thus cortisol biology during pregnancy is an important point of research^31,43^.

Although intimate partner violence during pregnancy is a worldwide-present phenomenon, it remains understudied^44,45^. Our study is among the first to evaluate the relation of IPV and maternal cortisol biology and their joint trajectory during pregnancy. Differentiating IPV from other stressors is essential for refining the concept of prenatal stress. The broad categorization of diverse exposures—such as psychiatric disorders, natural disasters, and adverse life events—under a single umbrella may obscure critical distinctions, potentially underestimating both the unique biological impacts of IPV and the opportunities for targeted interventions.

The association observed exclusively between acute exposure to physical or sexual violence by an intimate partner and lower cortisol levels aligns with emerging evidence suggesting reduced HPA-axis activity and reactivity in the context of chronic violence or extreme stress. Conceptualizing physical or sexual violence as the culmination of a broader pattern of sustained exposure to a violent environment, our findings suggest that intimate partner violence may exert distinct biological effects compared to other stressors. In this framework, acute exposure was linked to lower cortisol levels, whereas prior (chronic) exposure was associated with alterations in the expected cortisol trajectory of decrease in the postpartum.

Regarding the relationship between prenatal stress and cortisol, the lack of significant associations for most stress exposures contrasts with some previous studies that reported elevated cortisol levels in response to prenatal anxiety, depression, or perceived stress^46–48^. However, our findings align with the most recent meta-analyses on the topic showing inconsistent or weak associations between self-reported stress and cortisol levels^16,17^.

The absence of significant associations between most prenatal stress exposures and hair cortisol may be attributed to several factors. First, physiological adaptations during pregnancy may buffer cortisol responses to chronic stress^49^. Second, psychosocial and behavioral resilience factors could mitigate the impact of prenatal stress on cortisol regulation. Social support, coping mechanisms, and personal resources may dampen the physiological stress response, leading to an attenuation of cortisol elevations in response to chronic stress. Studies have shown that perceived social support during pregnancy can moderate HPA axis activity, potentially explaining the absence of strong cortisol differences in our sample^50,51^. The effects of stressors may be more complex than just raising or dampening cortisol levels, as different indexes are related to different factors^52^.

Finally, our use of binary measures for some stress exposures (e.g., presence vs. absence of intimate partner violence, anxiety, or depression) may have limited the ability to detect dose-dependent relationships between stress severity and cortisol levels. Future studies using more granular, continuous stress measures may better capture these associations.

Our findings raise important questions about the utility of cortisol as a biomarker of prenatal stress. While acute severe stressors, such as intimate partner violence, were associated with lower cortisol levels, most psychosocial stressors did not exhibit clear associations. This finding aligns with emerging evidence suggesting that cortisol may not be a universally reliable indicator of psychosocial stress^52,53^, and highlights the need for a more nuanced understanding of HPA axis regulation.

### Strengths and limitations

This study has several notable strengths. First, it utilized a well-characterized, population-based birth cohort with prospective data collection, reducing recall bias and enhancing temporal validity. Second, the use of hair cortisol as an objective biomarker allowed for the assessment of long-term cortisol exposure, rather than relying on single-time-point measures prone to diurnal variation. Third, multiple forms of prenatal stress were assessed, encompassing both acute and chronic exposures.

However, some limitations should be considered. The sample size was relatively small, potentially limiting statistical power to detect subtle associations. While hair cortisol provides a long-term measure of HPA axis activity, it may not be sensitive to short-lived stress responses that are better captured by salivary, sweat, or serum cortisol. At the same time, using cumulative averages of point measures provide theoretically the same information as hair cortisol with more precise temporal measurements and less prone to wash-out effects. Additionally, the binary classification of stress exposures may have obscured dose-response effects, warranting future studies with more nuanced assessments of stress severity and chronicity. Although intimate partner violence was associated with differences in hair cortisol levels, the number of exposed participants—particularly for physical or sexual violence during pregnancy—was relatively small. This limited the possibility of modeling exposure severity and may reduce statistical power and generalizability to populations with different prevalence or patterns of violence exposure.

## Conclusion

This study is among the first to examine the relationship between multiple prenatal stressors and maternal hair cortisol trajectories in a prospective cohort. Our findings suggest that while cortisol levels decline from pregnancy to postpartum, most prenatal stress exposures are not associated with maternal cortisol regulation, with the exception of acute intimate partner violence. These results underscore the complexity of HPA axis regulation during pregnancy and highlight the need for multifaceted approaches to studying prenatal stress and its biological consequences. Future research should explore additional biomarkers and integrate psychosocial resilience factors to enhance our understanding of the links between prenatal stress and maternal-fetal health.

## Supplementary Information

Below is the link to the electronic supplementary material.

Supplementary Material 1

## Data Availability

The data that support the findings of this study are derived from the BRISA birth cohort and contain sensitive personal and health information. Due to ethical and legal restrictions, the datasets are not publicly available. Access may be granted upon reasonable request to the corresponding author, subject to approval by the BRISA Cohort Steering Committee.
