# Supplementary material for: Prenatal stress, intimate partner violence and maternal cortisol trajectories: insights from a prospective birth cohort from São Paulo, Brazil
Source: Sci Rep. 2026 May 4;16:20585. doi: 10.1038/s41598-026-50811-9 (PMC13334050; doi:10.1038/s41598-026-50811-9)
Supplement: Supplementary file 1 — Supplementary Material 1 [file 41598_2026_50811_MOESM1_ESM.docx]

**SUPPLEMENTARY FILES**

**Supplementary Table S1. Percentage of missing data for study variables.**

| **Variable** | **N available** | **N missing** | **% missing** |
| --- | --- | --- | --- |
| Cortisol – 3M prepartum | 145 | 38 | 20.8 |
| Cortisol – 2M prepartum | 168 | 15 | 8.2 |
| Cortisol – 1M prepartum | 177 | 6 | 3.3 |
| Cortisol – Birth | 181 | 2 | 1.1 |
| Cortisol – 1M postpartum | 181 | 2 | 1.1 |
| Cortisol – 2M postpartum | 171 | 12 | 6.6 |
| Anxiety | 179 | 4 | 2.2 |
| Depression | 179 | 4 | 2.2 |
| Feelings About Pregnancy | 178 | 5 | 2.7 |
| (Acute) Prenatal Psychological Violence | 179 | 4 | 2.2 |
| (Acute) Prenatal Physical or Sexual Violence | 179 | 4 | 2.2 |
| Lifetime Psychological Violence | 179 | 4 | 2.2 |
| Lifetime Physical or Sexual Violence | 179 | 4 | 2.2 |

**Supplementary Table S2. Mean differences in hair cortisol concentrations between exposed and non-exposed groups at each time point.**

| **Exposure** | **Time point** | **Mean cortisol exposed** | **Mean cortisol unexposed** | **Mean difference** | **95% CI** |
| --- | --- | --- | --- | --- | --- |
| Anxiety | 3 months prepartum | 1.14 | 0.84 | 0.29 | -0.75 – 1.33 |
| Anxiety | 2 months prepartum | 0.85 | 0.93 | -0.07 | -1.06 – 1.20 |
| Anxiety | 1 month prepartum | 0.71 | 0.69 | 0.02 | -0.95 – 1.00 |
| Anxiety | Birth | 0.60 | 0.58 | 0.12 | -0.95 – 0.98 |
| Anxiety | 1 month postpartum | 0.41 | 0.41 | 0.01 | -0.93 – 0.95 |
| Anxiety | 2 months postpartum | 0.35 | 0.28 | 0.07 | -0.89 – 1.03 |
| Depression | 3 months prepartum | 1.14 | 0.73 | 0.40 | -0.78 – 1.59 |
| Depression | 2 months prepartum | 0.89 | 0.61 | 0.27 | -0.89 – 1.44 |
| Depression | 1 month prepartum | 0.71 | 0.66 | 0.05 | -1.12 – 1.23 |
| Depression | Birth | 0.60 | 0.56 | 0.04 | -1.06 – 1.13 |
| Depression | 1 month postpartum | 0.41 | 0.50 | -0.09 | -1.16 – 0.98 |
| Depression | 2 months postpartum | 0.34 | 0.40 | -0.06 | -1.11 – 0.99 |
| Feelings About Pregnancy | 3 months prepartum | 1.16 | 0.91 | 0.25 | -0.57 – 1.08 |
| Feelings About Pregnancy | 2 months prepartum | 0.99 | 0.59 | 0.40 | -0.35 – 1.15 |
| Feelings About Pregnancy | 1 month prepartum | 0.85 | 0.39 | 0.47 | -0.27 – 1.20 |
| Feelings About Pregnancy | Birth | 0.79 | 0.18 | 0.61 | -0.10 – 1.32 |
| Feelings About Pregnancy | 1 month postpartum | 0.57 | 0.09 | 0.48 | -0.21 – 1.17 |
| Feelings About Pregnancy | 2 months postpartum | 0.45 | 0.13 | 0.32 | -0.38 – 1.01 |
| (Acute) Prenatal Psychological Violence | 3 months prepartum | 1.21 | 0.68 | 0.53 | -0.33 – 1.41 |
| (Acute) Prenatal Psychological Violence | 2 months prepartum | 0.92 | 0.61 | 0.31 | -0.53 – 1.15 |
| (Acute) Prenatal Psychological Violence | 1 month prepartum | 0.77 | 0.44 | 0.33 | -0.50 – 1.16 |
| (Acute) Prenatal Psychological Violence | Birth | 0.63 | 0.44 | 0.19 | -0.62 – 1.01 |
| (Acute) Prenatal Psychological Violence | 1 month postpartum | 0.38 | 0.56 | -0.18 | -0.98 – 0.62 |
| (Acute) Prenatal Psychological Violence | 2 months postpartum | 0.29 | 0.57 | -0.28 | -1.09 – 0.54 |
| (Acute) Prenatal Physical or Sexual Violence | 3 months prepartum | 1.23 | 0.09 | 1.13 | 0.03 – 2.24 |
| (Acute) Prenatal Physical or Sexual Violence | 2 months prepartum | 0.97 | -0.10 | 1.07 | -0.02 – 2.17 |
| (Acute) Prenatal Physical or Sexual Violence | 1 month prepartum | 0.80 | -0.09 | 0.90 | -0.18 – 1.97 |
| (Acute) Prenatal Physical or Sexual Violence | Birth | 0.69 | -0.18 | 0.86 | -0.21 – 1.93 |
| (Acute) Prenatal Physical or Sexual Violence | 1 month postpartum | 0.45 | 0.06 | 0.40 | -0.67 – 1.46 |
| (Acute) Prenatal Physical or Sexual Violence | 2 months postpartum | 0.39 | -0.14 | 0.53 | -0.58 – 1.64 |
| Lifetime Psychological Violence | 3 months prepartum | 1.30 | 0.38 | 0.91 | 0.05 – 1.78 |
| Lifetime Psychological Violence | 2 months prepartum | 1.00 | 0.29 | 0.72 | -0.13 – 1.56 |
| Lifetime Psychological Violence | 1 month prepartum | 0.83 | 0.21 | 0.61 | -0.23 – 1.45 |
| Lifetime Psychological Violence | Birth | 0.70 | 0.20 | 0.50 | -0.32 – 1.32 |
| Lifetime Psychological Violence | 1 month postpartum | 0.47 | 0.26 | 0.21 | -0.60 – 1.02 |
| Lifetime Psychological Violence | 2 months postpartum | 0.36 | 0.34 | 0.02 | -0.81 – 0.85 |
| Lifetime Physical or Sexual Violence | 3 months prepartum | 1.22 | 0.042 | 1.18 | 0.048 – 2.31 |
| Lifetime Physical or Sexual Violence | 2 months prepartum | 0.97 | -0.10 | 1.07 | -0.02 – 2.16 |
| Lifetime Physical or Sexual Violence | 1 month prepartum | 0.81 | -0.19 | 1.00 | -0.07 – 2.07 |
| Lifetime Physical or Sexual Violence | Birth | 0.70 | -0.28 | 0.97 | -0.09 – 2.04 |
| Lifetime Physical or Sexual Violence | 1 month postpartum | 0.47 | -0.09 | 0.56 | -0.51 – 1.62 |
| Lifetime Physical or Sexual Violence | 2 months postpartum | 0.41 | -0.29 | 0.71 | -0.37 – 1.78 |
